# Evaluation of tectonically enhanced radon in fault zones by quantification of the radon activity index

**DOI:** 10.1038/s41598-022-26124-y

**Published:** 2022-12-14

**Authors:** Eleonora Benà, Giancarlo Ciotoli, Livio Ruggiero, Chiara Coletti, Peter Bossew, Matteo Massironi, Claudio Mazzoli, Volkmar Mair, Corrado Morelli, Antonio Galgaro, Pietro Morozzi, Laura Tositti, Raffaele Sassi

**Affiliations:** 1 Dipartimento di Geoscienze, Università di Padova, Via Gradenigo 6, 35131 Padova, Italy; 2 Istituto di Geologia Ambientale e Geoingegneria (IGAG), Consiglio Nazionale delle Ricerche (CNR), 00015 Monterotondo, Rome, Italy; 3 Istituto Nazionale di Geofisica e Vulcanologia (INGV), Via di Vigna Murata 605, 00143 Rome, Italy; 4 Retired from Federal Office for Radiation Protection (BfS), Section Radon and NORM, Köpenicker Allee 120-130, 10318 Berlin, Germany; 5 Provincia Autonoma di Bolzano, Ufficio Geologia e Prove Materiali, Cardano-Kardaun, Italy; 6 Dipartimento di Chimica “G. Ciamician”, Università di Bologna, Via Selmi 2, 40126 Bologna, Italy

**Keywords:** Environmental sciences, Natural hazards, Solid Earth sciences

## Abstract

This work highlights the importance of the Geogenic Radon Potential (GRP) component originated by degassing processes in fault zones. This Tectonically Enhanced Radon (TER) can increase radon concentration in soil gas and the inflow of radon in the buildings (Indoor Radon Concentrations, IRC). Although tectonically related radon enhancement is known in areas characterised by active faults, few studies have investigated radon migration processes in non-active fault zones. The Pusteria Valley (Bolzano, north-eastern Italy) represents an ideal geological setting to study the role of a non-seismic fault system in enhancing the geogenic radon. Here, most of the municipalities are characterised by high IRC. We performed soil gas surveys in three of these municipalities located along a wide section of the non-seismic Pusteria fault system characterised by a dense network of faults and fractures. Results highlight the presence of high Rn concentrations (up to 800 kBq·m^−3^) with anisotropic spatial patterns oriented along the main strike of the fault system. We calculated a Radon Activity Index (RAI) along north–south profiles across the Pusteria fault system and found that TER is linked to high fault geochemical activities. This evidence confirms that TER constitutes a significant component of GRP also along non-seismic faults.

## Introduction

Radon (^222^Rn) is considered the dominant source of human exposure to ionizing radiation. Being a natural radioactive gas, epidemiological studies provided evidence for a marked increased risk of lung cancer associated with long-term exposure also to relatively low Indoor Radon Concentrations (IRC), as well as to its decay products^1^. As a consequence, the World Health Organization (WHO) classified radon as the second leading cause of lung cancer after cigarette smoking^2^. IRC in buildings is the main target variable of current regulations^3,4^; however, this parameter is controlled by soil-gas radon concentration, which is commonly assumed to be primarily correlated to the amount of uranium content in soils and rocks (geogenic radon; GR). GR is usually quantified by the Geogenic Radon Potential (GRP) that represents what “Earth delivers” in terms of radon available to enter buildings, and it can be considered as an indicator of the susceptibility of an area to geogenic radon^5^. Additionally, IRC strongly depends on anthropogenic factors, namely building characteristics and usage patterns.

GR is the result of two main components: Rn from the source, e.g. the radon produced from the natural radioactive decay of radionuclides in rocks, soil and groundwater (background); and migrated Rn, e.g. the radon derived from diffusion and migration processes in the subsurface, and their influence factors, mainly occurring along more permeable pathways, i.e. faults, fractures and cavities (e.g., in karst areas). Both components contribute to the amount of Rn in soil potentially available (e.g., GRP) to exhale from the ground and infiltrate into buildings^6^.

Radon transport over distance in fault zones may locally represent a significant additional contribution to the Rn background originated by radionuclide decay in the source rocks; this quantity can be defined as Tectonically Enhanced Radon (TER).

TER measures the “strength” of the tectonic factor that can potentially increase the radon originated from the local lithology (and, as a consequence, can affect the potential increase of the IRC), due to:

- the action of carrier gases (e.g. CO_2_, CH_4_) migrating from deep sources by advection along faults^7^.
- the increase of rock permeability in the damage zones enveloping active faults^8–14^.

**Figure 1:**
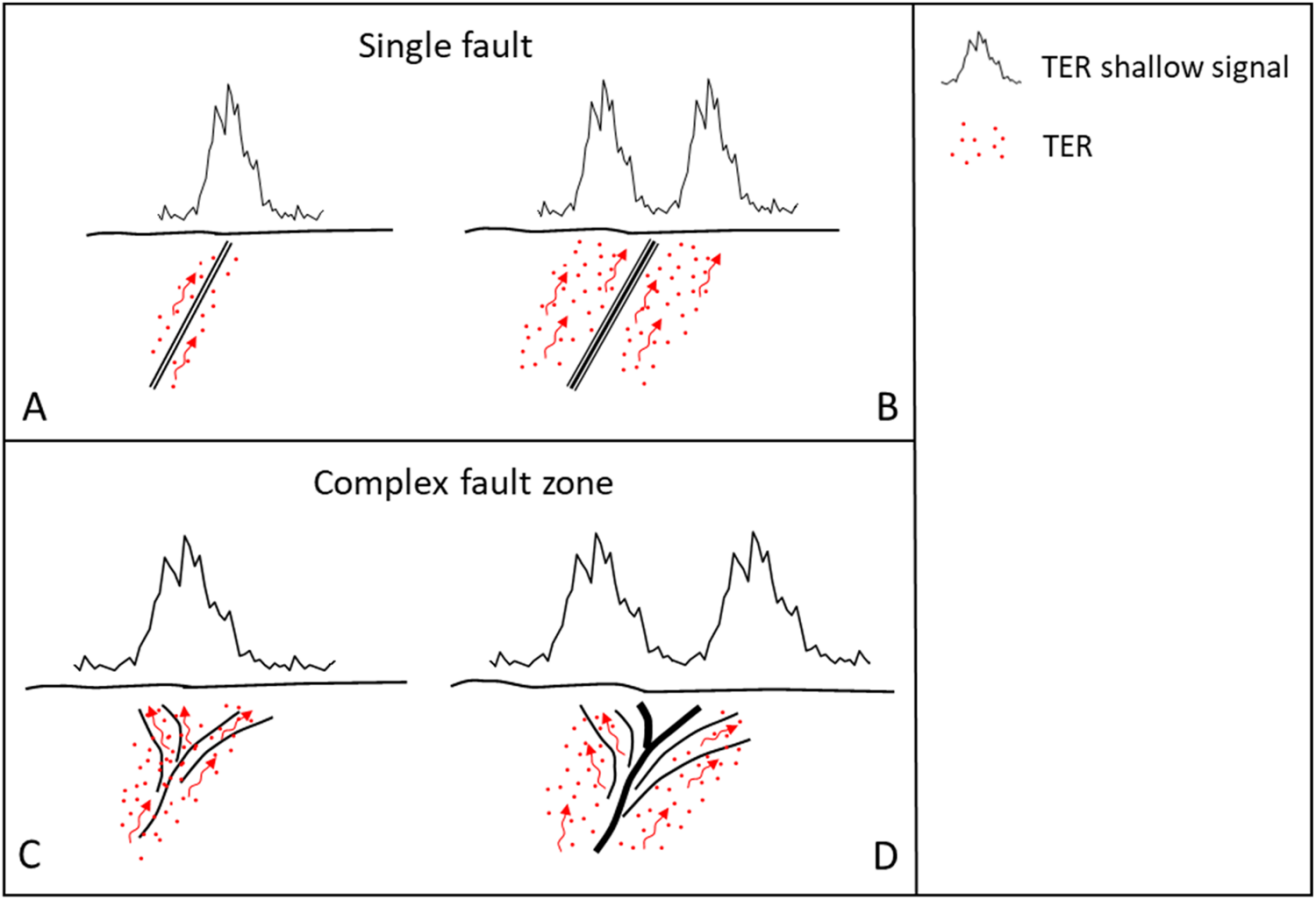
The figure represents the two different cases of a single fault and complex fault zone: (**A**) single fault with permeable core and damage zone, TER shallow signal is represented by a single-peak anomaly; (**B**) single fault with non-permeable core and wide and permeable damage zone, TER signal may show a double-peak anomaly; (**C**) complex fault zone with permeable core and damage zone, TER signal occurs as a single-peak; (**D**) complex fault zone with non-permeable core, and wide and permeable damage zone; TER is represented by a double-peak signal.

Numerous studies report examples of radon migration along faults that may be ascribed to the TER concept but specific studies aimed at quantifying this geogenic component of radon are missing. However, most of these studies deal with seismic faults, in contrast to the aseismic fault system discussed in our work. Both seismic and aseismic faults can still be expected to have certain features in common; while aseismic faults can be supposed to lack the temporal dimension of seismic faults which lead to temporally variable gas transport and exhalation. This effect is used for diagnosis and forecast in volcanology and seismic science, but not further discussed here. For example, Ciotoli et al.^7^ investigated radon distribution in the Fucino Plain (central Italy), a tectonically active intermontane basin bordered and crossed by a complex network of deeply buried and/or shallow faults and fractures characterized by high seismic activity (e.g., magnitude 7.0, Avezzano earthquake of 13 January 1915^15^). In this area, radon anomalies up to 5 times the background soil production (25 kBq·m^−3^), occur along the main faults of the basin even when buried under thick sedimentary covers (up to 900 m). Fault-related anisotropy affects radon distribution at the surface, and provides a clear correlation between the shape and orientation of radon anomalies and the geometry of the recognised damage zones in the area. Seminsky et al.^11^ studied the variations of radon activity in the crustal fault zone of the Baikal-Mongolian seismic belt and revealed that the radon anomalies occurring along the investigated profiles crossing the Khustai fault are five times higher than the background value (3.9 kBq·m^−3^). Wang et al.^16^ found radon concentrations in soil gas eight times higher than the background value (4.7 kBq·m^−3^) along the active Tangshan fault (Northern China). In particular, radon enhanced values in the area showed close relation with the seismic activity along fault zones. Active segments of faults and associated damage zones along which larger earthquakes nucleated may act as preferential paths for enhanced radon migration. These studies suggest that the anisotropic spatial distribution of radon anomalies along fault zones is strictly related to their orientation, geometrical complexity (i.e. single fault density vs complex fault system), seismic activity and the relation between width of the core zone and extension of the damage zone (e.g., presence of transverse faults, i.e. relay ramp^17^) (Fig. 1).

**Figure 2:**
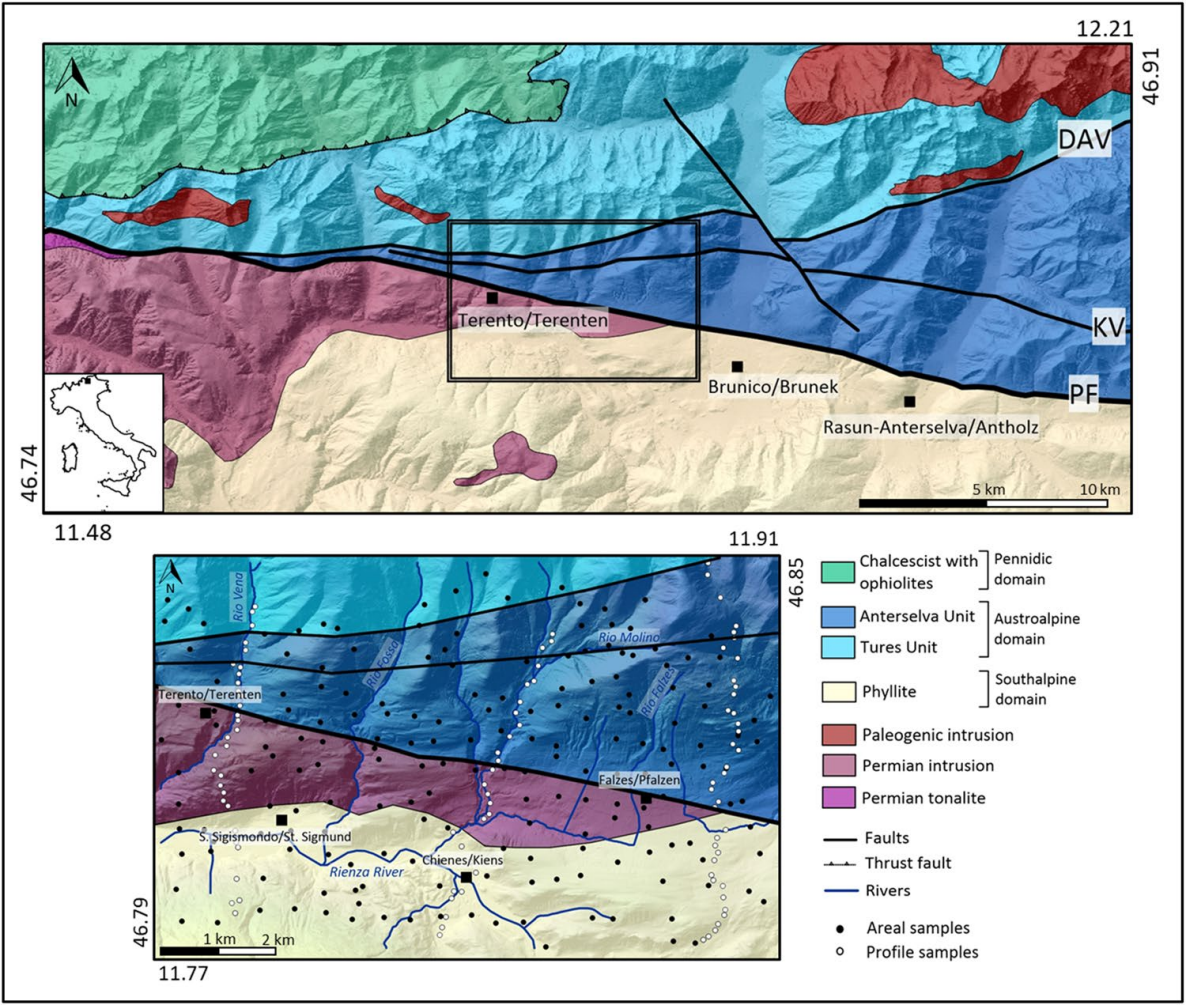
Geological sketch map of the Eastern Alps (**a**) (modified from^21^). The study area shows the location of the soil gas samples: areal samples (in black); and profiles samples (P1, P2, P3, in white). PF = Pusteria/Pustertal fault, KV = Kalkstein-Vallarga/Weitental fault^19^, DAV = Deffereggen-Anterselva/Antholz-Valles/Vals mylonitic zone.

In this work, we focus on the analysis of the distribution and the magnitude of radon, thoron and CO_2_ soil gas anomalies in the Pusteria/Pustertal Valley (north-eastern Italy, Fig. 2). This area is characterised by the Periadriatic fault system which is the aseismic tectonic boundary between the Austroalpine orogenic wedge and the Southalpine indenter in the Eastern Alps. In the study area, this non-seismic fault system is composed of three main E-W trending faults: (i) the Pusteria/Pustertal fault (PF), a sub vertical fault system that accommodated a dextral transpressive kinematics during the Alpine orogenesis since late Oligocene^18–21^; (ii) the Kalkstein-Vallarga/Weitental fault (KV), a minor fault still pertaining to the same Periadriatic fault system^22^; (iii) the Deffereggen-Anterselva/Antholz-Valles/Vals line (DAV), a mylonitic shear zone marking the southern boundary of the Alpine (Paleogenic) metamorphic overprint within the Austroalpine basement^23^. In the study area, the outcropping crystalline basement is composed of orthogneiss and paragneiss (Austroalpine domain) to the north of the PF, and phyllites intruded by granites (Southalpine domain) to the south of the PF (Fig. 2).

The main objectives of our work are: (i) to evaluate the potential degassing processes along a non-seismically active fault (i.e., the Pusteria fault system), (ii) to quantify TER in terms of a Radon Activity Index (RAI) and iii) to compare RAI magnitude with those calculated for seismically active fault systems from the literature. The obtained results highlight that TER may constitute an important additional component to GRP also along aseismic fault systems, thus potentially enhancing influx of radon into buildings.

## Results

^222^Rn and thoron (^220^Rn) activities, CO_2_ and O_2_ concentrations were measured directly in the field at 278 sites according to a regular grid and along three N-S profiles crossing the aseismic Pusteria fault system, over an area of about 60 km^2^.

Table 1 shows descriptive statistics (i.e., main numerical indexes) of all the studied gases. The significance of Kolmogorov–Smirnov test (p < 0.01) confirms the non-normal distribution of all the variables (measured on the grid and along the three profiles), especially those of  ^222^Rn, ^220^Rn and CO_2_ which showed a skewed distribution (see Supplementary Fig. S1a).

**Table 1:** Descriptive statistics of soil gas data.

	Soil data	N	Mean	CI − 95%	CI + 95%	GM	Min	Max	LQ	UQ	Std.Dev	SK	Median
A	^222^Rn	278	67.44	55.15	79.74	35.16	1.54	815.96	15.36	78.61	104.11	4.46	36.52
	^220^Rn	272	17.60	16.21	18.98	14.55	1.04	102.07	9.78	22.82	11.62	2.70	15.46
	CO_2_	277	2.51	2.24	2.79	1.78	0.20	16.00	1.00	3.40	2.30	2.13	1.71
	O_2_	236	18.06	17.70	18.43	17.71	3.10	20.60	17.30	19.85	2.85	− 2.43	19.10
P1	^222^Rn	17	55.78	20.33	91.22	29.74	7.65	253.24	10.33	53.78	68.94	1.84	24.73
	^220^Rn	17	21.00	15.95	26.05	18.57	5.70	37.02	14.59	30.88	9.81	0.18	20.59
	CO_2_	17	2.79	1.41	4.17	2.03	0.60	10.80	1.00	3.20	2.68	2.15	1.80
	O_2_	17	17.76	16.14	19.38	17.38	7.60	20.20	16.80	19.80	3.15	− 2.42	19.10
P2	^222^Rn	21	66.10	45.37	86.83	50.88	13.71	186.20	33.54	87.39	45.53	0.97	54.51
	^220^Rn	20	18.19	13.54	22.85	15.79	5.23	41.66	10.97	22.17	9.95	1.02	15.92
	CO_2_	21	2.89	2.19	3.59	2.43	0.60	6.20	1.40	3.80	1.54	0.32	3.00
	O_2_	21	17.29	16.24	18.34	17.13	12.10	20.20	16.40	19.10	2.31	− 0.84	17.50
P3	^222^Rn	24	68.83	24.63	113.04	33.80	6.76	448.76	16.05	75.11	104.69	2.68	27.54
	^220^Rn	23	12.44	8.34	16.54	9.79	1.04	47.54	6.61	15.74	9.48	2.43	9.75
	CO_2_	24	1.68	1.11	2.25	1.29	0.30	5.80	0.80	2.30	1.35	1.79	1.20
	O_2_	24	19.15	18.50	19.80	19.08	13.20	20.40	18.90	20.05	1.53	− 2.83	19.65

A = Total dataset; P1, P2, P3 = profiles; *N *Number of samples; Mean; *CI* (−95%—+ 95%) Confidence interval of the mean; *GM* Geometric mean; *Min* Minimum value; *Max* Maximum value; *LQ* Lower quartile; *UQ* Upper quartile; *Std.Dev.* Standard deviation; *SK* Skewness; Median.

Descriptive statistics on the total dataset (A) highlights that ^222^Rn ranges between 1.54 and 815.96 kBq·m^−3^. We considered the similarity between median (36.52 kBq·m^−3^) and geometric mean (GM) (35.16 kBq·m^−3^) as a representative indicator of the approximately log-normal distribution of the variables, since median is not influenced by the presence of outliers and GM is the mean value of the log-transformed data. ^220^Rn values range between 1.04–102.07 kBq·m^−3^ with a median of 15.46 kBq·m^−3^ and a GM of 14.55 kBq·m^−3^. CO_2_ measurements range between 0.20% v/v and 16.00% v/v with a median of 1.71% v/v and GM equal to 1.78% v/v.

Descriptive statistics of the three profiles showed that ^222^Rn varies between 7.65–253.24 kBq·m^−3^ (P1), 13.71–186.20 kBq·m^−3^(P2) and 6.76–448.76 kBq·m^−3^ (P3). The GM values are 29.74 kBq·m^−3^ (P1), 50.88 kBq·m^−3^ (P2) and 33.80 kBq·m^−3^ (P3), respectively.

^220^Rn values range 5.70–37.02 kBq·m^−3^ (P1), 5.23–41.66 kBq·m^−3^ (P2) and 1.04–47.54 kBq·m^−3^ (P3). The GM values are 18.57 kBq·m^−3^ (P1), 15.79 kBq·m^−3^ (P2) and 9.79 kBq·m^−3^ (P3).

CO_2_ concentrations range between 0.60–10.80% v/v (P1), 0.60–6.20% v/v (P2), and 0.30%-5.80% v/v (P3). The respective GM are equal to 2.03% v/v (P1), 2.43% v/v (P2) and 1.29% v/v (P3).

It is worth noting that maximum ^222^Rn value (815.96 kBq·m^−3^), as well as values measured along the three profiles (253.24 kBq·m^−3^, 186.20 kBq·m^−3^ and 448.76 kBq·m^−3^ for P1, P2 and P3, respectively) are among the highest values measured in active tectonic areas in Italy, based on more than 30,000 data collected in Central and Southern Italy^25^.

The normal probability plots (see supplementary materials, Fig. S1b) describe both the background values and the anomaly thresholds of the different gas species: 50 kBq·m^−3^ for ^222^Rn, 15 kBq·m^−3^ for ^220^Rn and 4% v/v for CO_2._

We applied the Akerblom formula^26^ (see section on Radionuclide content in Methods) to calculate the radon at equilibrium with the activity concentration of the ^226^Ra (its direct parent radionuclide) in the main outcropping lithologies. The formula provides ^222^Rn mean values of 83.5 kBq·m^−3^ for the Austroalpine gneiss, 68.3 kBq·m^−3^ for the Permian granite and 27.7 kBq·m^−3^ for the Southalpine phyllite, respectively (see Supplementary Table S1). The mean value of 59.8 kBq·m^−3^ agrees with that obtained by the NPP (Normal Probability Plots; see Supplementary Fig. S1b).

Variogram analysis has been performed on ^222^Rn, ^220^Rn and CO_2_ data which were log-transformed to minimize the effect of outliers^27–29^. The experimental variograms were calculated along four directions to highlight the presence of anisotropies (e.g., fault related gas anomalies) in the spatial distribution of the data. In general, all gases showed a clear anisotropic behaviour along the E-W direction. The experimental variograms calculated along the maximum axes of the anisotropy ellipse showed correlations consistent with the main directions of faults and fractures. In particular, the main anisotropy directions are about 90° for ^222^Rn, 50° for ^220^Rn and 95° for CO_2_ (see Supplementary Fig. S2). Variogram models were used within the ordinary kriging algorithm to predict values at unsampled locations and construct gas distribution maps (see section on Statistical and Geospatial analyses in Methods).

**Figure 3:**
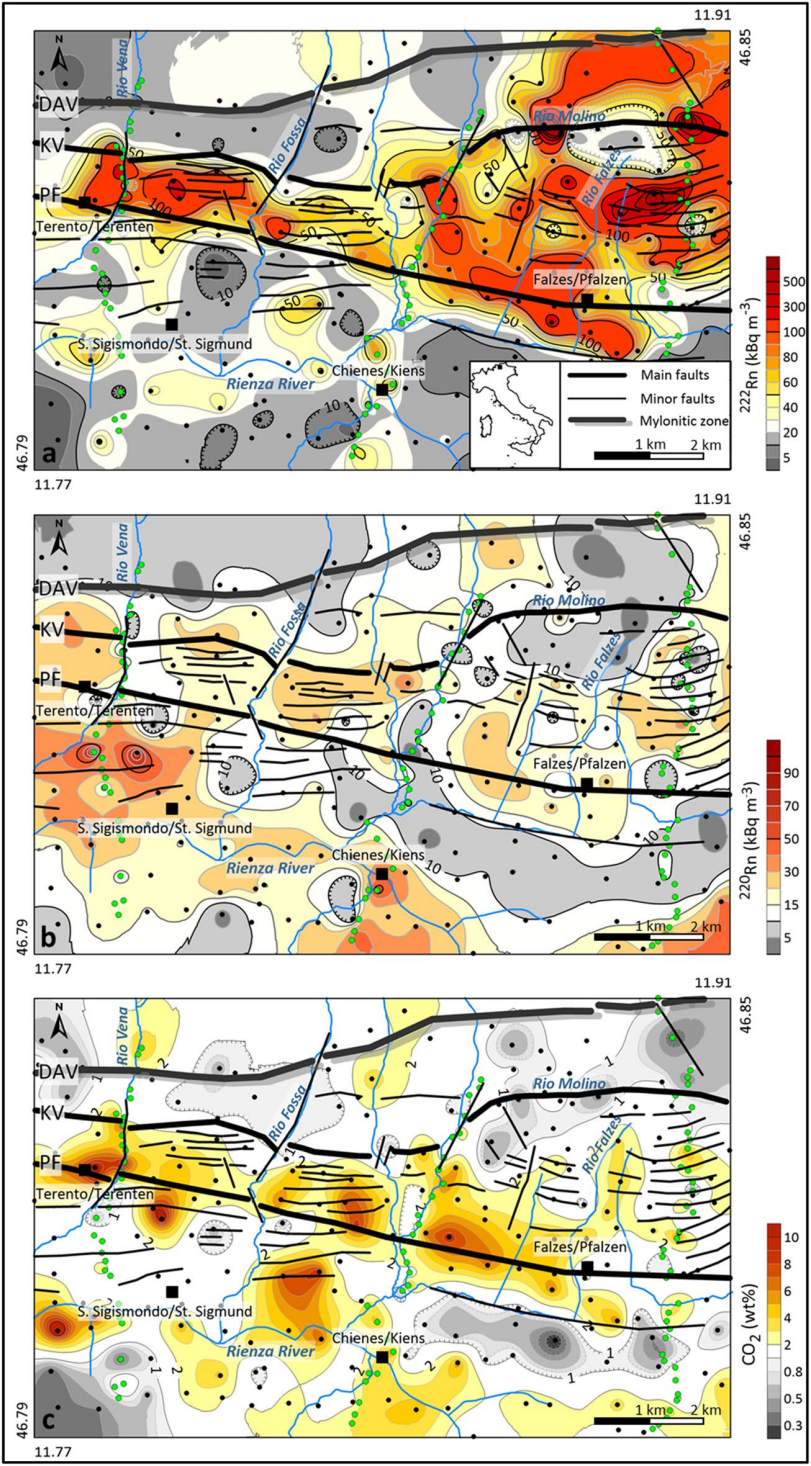
Contour maps of (**a**) ^222^Rn, (**b**) ^220^Rn and (**c**) CO_2_ of the study area. In the maps the structural model of the study area is also reported, with the interpreted structures divided into: main faults (PF = Pusteria/Pustertal Fault and KV = Kalkstein-Vallarga/Weitental fault); minor faults; Mylonitic zone (DAV = Deffereggen-Anterselva/Antholz-Valles/Vals mylonitic zone). The coloured points represent the location of the soil-gas measurements: areal sampling in black and the three profiles (P1, P2, P3) in green.

According to the maximum anisotropy axis discussed above, ^222^Rn anomalies well fit the E-W trend of the Periadriatic system from the DAV line, to the north, to the PF, to the south (Fig. 3a). This trend also follows the direction of the brittle fracture zone between the main faults, which includes conjugate minor faults and fractures roughly parallel to the main faults^30,31^. The highest anomalous values (> 100 kBq·m^−3^ and up to 800 kBq·m^−3^) occur in the eastern sector (municipality of Falzes/Pfalzen). This area shows the highest radon values up to 815.96 kBq· m^−3^. In contrast, the plain area south of the PF displays the lowest radon values, below the background (< 50 kBq·m^−3^).

The lowest ^220^Rn values (< 30 kBq·m^−3^) have been measured in the fracture zone between the DAV and PF, while the highest ones occur to the south of PF corresponding to the western sector (Terento/Terenten municipality) (Fig. 3b).

The distribution of CO_2_ anomalies (> 4% v/v) also follows the E-W trend of the PF (maximum anisotropy axis = 95°) (Fig. 3c). The area south of the PF shows a CO_2_ anomaly greater than 4% in the municipality of Chienes/Kiens; in contrast, the easternmost sector of the study area only reports low CO_2_ concentration values.

In general, the most fractured sector between DAV and PF shows higher values of ^222^Rn with respect to neighbouring zones. It is worth noting that ^222^Rn and CO_2_ anomalies coincide along the PF, whereas CO_2_ displays high values also south of the PF.

## Discussion

We performed morphological analysis from the DTM (2.5 × 2.5 m Digital Terrain Model) in order to reconstruct a structural model of the area (see section on Structural Model in Methods). The model highlights a series of minor faults and fractures located among the main faults in the area (PF, KV line and DAV). This structural network includes the potentially permeable pathways for gas advection.

As widely reported in the literature, radon generated by ^238^U decay in rocks and soil can escape by diffusion from the surface and accumulate/disperse at shallow depth in soils, or migrate by advection upwards at greater distance from deeper sources along preferential pathways, such as faults and fractures, transported by gas carriers (CO_2_, CH_4_, etc.)^7,25,32–34^. Meteorological parameters (e.g., pressure, humidity, temperature) can also affect the distribution of radon in shallow soil, but they cannot explain the sharp anisotropy of the radon distribution along the fault strike^35,36^.

In this study area, ^222^Rn and CO_2_ anomalies generally well fit with the main orientation of the Periadriatic fault system. However, despite both gases show the same anisotropy ratio (R = 2), as observed in the experimental variograms (see Supplementary Fig. S2), ^222^Rn anomalies show higher spatial continuity compared to CO_2_. As a result, the spatial distribution of ^222^Rn and CO_2_ concentrations show different patterns characterised by elongated anomalies, and aligned spot anomalies, respectively, as also elsewhere observed by Ciotoli et al.^7^.

The extent and the elongation of radon anomalies spatially changes from a sharp (narrowly elongated anomalies) pattern in the western sector (from Terento/Terenten to Chienes/Kiens municipalities) to a more diffuse pattern in the eastern sector (Falzes/Pfalzen municipality), where the KV is displaced by secondary orthogonal NNE-SSW trending faults and width of the fractured area between KV and PF increases. It is worth noting that the distance (≈ 2 km) among these secondary faults is consistent with the range of the experimental variogram of the radon data measured along the major axis of the anisotropy ellipse. This parameter represents the distance at which Rn migration along the fault system shows a continuous behaviour, and thus indicates the extension of the permeable sectors along the PF system that can be dissected by minor faults according to a series of relay ramps that may interrupt the continuity of this tectonic system. Accordingly, the length of the minimum anisotropy axis (≈ 1000 m) well fit with the N-S extension of the elongated radon anomaly occurring from Terento/Terenten to Chienes/Kiens municipalities. Furthermore, though CO_2_ anomalies show a spotty distribution pattern, they are also consistent with the orientation of the PF and confirms the role of CO_2_ as carrier gas for ^222^Rn from deeper sources. A scatterplot between radon and CO_2_ values (see Supplementary Fig. S3), occurring in correspondence of the anomalous area along the Pusteria Fault, shows that there is a significant linear relationship between the two gas species (*p* value < 0.0001) with coefficients R = 0.723 and R^2^ = 0.523. This result confirms that CO_2_ acts as a carrier gas for radon along the main fault. In the southern sector (Chienes/Kiens municipality and S. Sigismondo/St. Sigmund village), low ^222^Rn (below the background value of 50 kBq m^−3^) and high ^220^Rn activities suggest the presence of high permeable soils in the shallow environment, considering the very short half-life of ^220^Rn (55.6 s). According to this hypothesis, in this area CO_2_ anomalies can be linked to shallower factors (e.g., soil humidity, organic matter) caused by the presence of the Rienza/Rienz river and farmed fields.

The eastern sector (Falzes/Pfalzen municipality) displays the highest ^222^Rn activities (from 100 to 800 kBq·m^−3^) measured in the area; these values are comparable with maximum ^222^Rn activity (order of magnitude 10^2^ kBq·m^−3^) measured in different seismic intermontane basins in Italy^25^ and in China^13^. In this sector, ^222^Rn anomalies show a more diffuse pattern though they are also characterised by local E-W anisotropy due to the presence of minor faults and according to the direction of the main fault system. In contrast, CO_2_ spatial distribution does not show anomalous concentrations. This might be due to an increase in soil ventilation (i.e., CO_2_ dilution) caused by the higher elevation of this zone (> 1800 m.a.s.l.), the reduced soil thickness and rock weathering^36^ (i.e., the diffused presence of slope deposits consisting of large loose rock blocks). However, all these factors do not seem to affect ^222^Rn emission in terms of TER.

In contrast, in the western sector (Terento/Terenten municipality) ^222^Rn anomalies show a more defined anisotropic pattern extending towards the central sector (Chienes/Kiens municipality) where the ^222^Rn anomalies are mainly restricted between the PF and the KV. However, this area is characterised by a less pervasive large-scale fracturing and thus radon measurements show lower values than those measured in the eastern sector (mean ^222^Rn activities of 75 kBq·m^−3^ and 130 kBq·m^−3^, respectively).

Despite the calculated mean value of ^222^Rn activity at equilibrium with ^226^Ra is slightly higher than that observed in the NPP, the contribution of the lithology is not high enough to justify the magnitude of the anomalies measured in the eastern sector (Falzes/Pfalzen area) and along the whole Pusteria fault. Therefore, we can conclude that all values above the anomaly threshold of 50 kBq·m^−3^ are reasonably supported by an upward migration along the fracture zone associated to the PF that promotes tectonically enhanced radon at the surface thus contributing to the increase of the GRP in terms of TER.

Based on the above considerations, we can confirm that the study area shows different pattern of ^222^Rn, CO_2_ and ^220^Rn spatial distribution of the gas anomalies at the surface, primarily related to the extension of the fracture zone and the length of the dislocation, and secondarily to local geological factors. These parameters mainly govern the pattern and the magnitude of the observed fault-linked Rn anomalies, thus suggesting a different geochemical activity (i.e., the gas-bearing property) of the fault segments that makes up the PF system^10,11^.

In order to evaluate the correlation between TER and geometry of the PF system, we firstly investigated the relationship between radon anomalies and fault/fracture density map obtained by Kernel density algorithm (see section on Structural Model in Methods) in order to identify TER zones. Then we tried to quantify the TER zones in terms of radon activity index (RAI) (see section on RAI Calculation in Methods)^11^.

**Figure 4:**
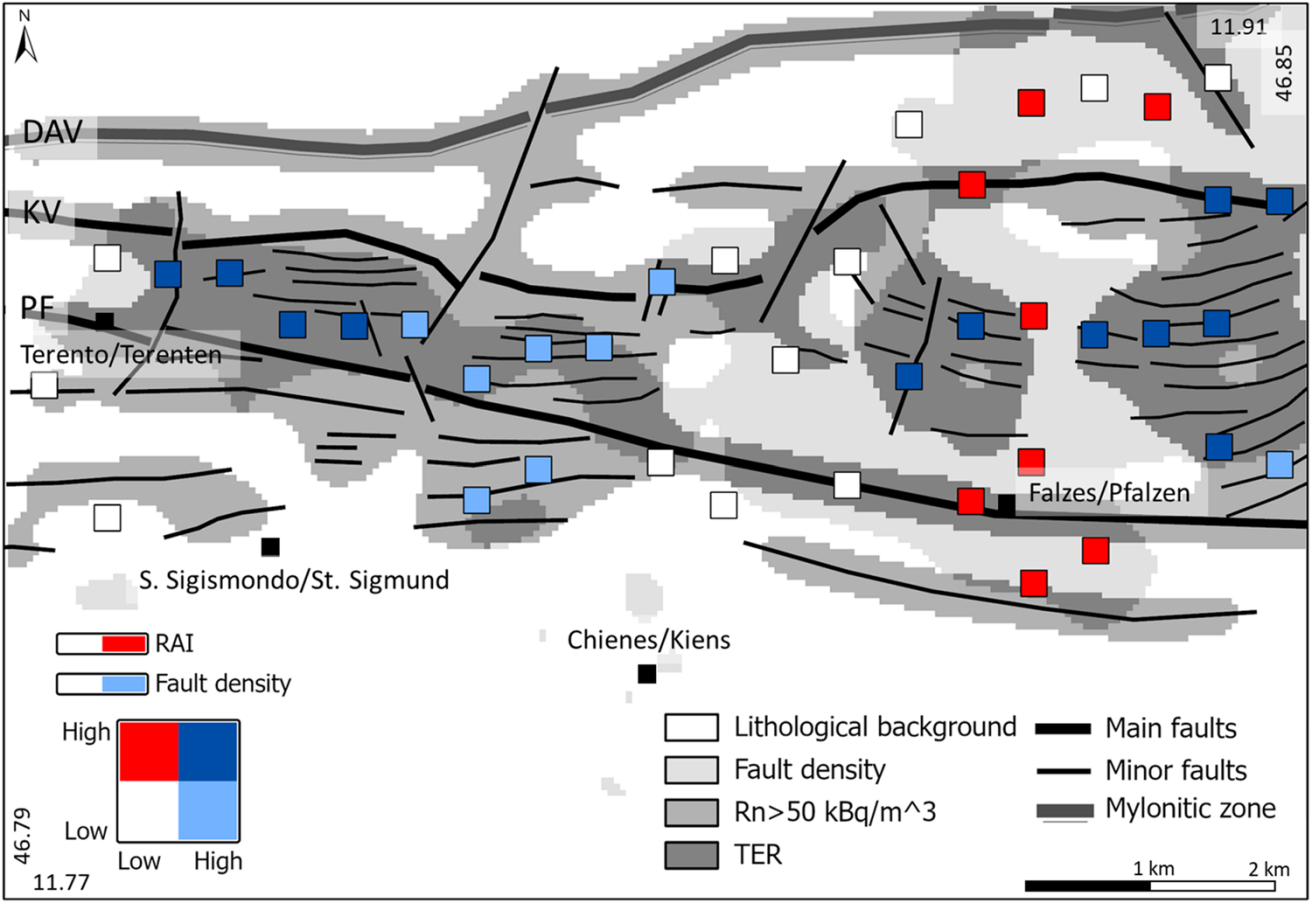
Summary sketch map of the areas characterised by radon fault-linked anomalies. The figure shows four different zones: (i) the lithological background (white area); (ii) the fault density (pale grey area); (iii) the area with radon activity higher than the background (> 50 kBq m^−3^) (silver grey area); (iv) the zones with high fault-linked radon values (e.g., TER) (dark grey areas). The coloured square represents the location of RAI values related to the profile peaks and have been divided as follows: (i) white squares: low RAI value, low fault density; (ii) light-blue squares: low RAI value, high fault density; (iii) red squares: high RAI values, low fault density; (iv) blue squares: high RAI values, high fault density (strictly related to the TER zones). PF = Pusteria/Pustertal fault; KV = Kalkstein-Vallarga/Weintal fault; DAV = Deffereggen-Anterselva/Antholz-Valles/Vals mylonitic zone.

Figure 4 shows a summary sketch map of the areas characterised by fault-linked Rn anomalies (e.g., TER). The map was obtained by reclassifying and overlaying radon anomalies (above 50 kBq·m^−3^) and fault/fracture density map (> 1.5 fault/km^2^). The figure also highlights the distribution of the main and minor faults reported in the structural model (see section on Structural Model in Methods).

In particular, the map shows 4 zones characterised by different radon sources and magnitude:

- the zone of lithological background (white area), statistically and geochemically defined by radon values below 50 kBq·m^−3^; this zone (about 34 km^2^) is characterised by Rn mean value of 20.5 kBq·m^−3^;
- the zone of high fault/fracture density (> 1.5 fault/km^2^) but with radon values below the background (pale grey area); this zone (about 14 km^2^), where the presence of non-permeable fault segments is supposed, is characterised by Rn mean value of 22.3 kBq·m^−3^
- the zone with radon activity higher than the background (silver grey area); this area, apparently not correlated to zones with high fault density, mostly occurs in the fractured area recognised within the fault system. This zone (about 8 km^2^) is characterised by high Rn mean value of 111.5 kBq·m^−3^ that suggests the presence of radon advection processes which change toward the surface in a more diffusive (even laterally) character; this may explain the more pervasive distribution of radon anomalies;
- the TER zone (about 12 km^2^) (dark grey area) is characterised by very high fault-linked radon anomalies with mean value of 154.0 kBq·m^−3^ mainly occurring in the area comprised between the KV and PF.

As TER represents the radon contribution from faulted areas, the quantitative evaluation of the contrast between the magnitude of the soil-radon anomalies (Rn_Max_) near the fault and the lithological background (Rn_BG_) was carried out by calculating the RAI for each fault-related radon concentration peak recognised along 21 N-S profiles (P4-P24) extrapolated from the estimated Rn grid map (Fig. 3a). The ratio between these two values can be considered as a function of the geochemical activity of the fault, as well as of the degree of fracturing in the damage zone. In general, high RAI values correspond to high Rn absolute concentration values in the soil-gas. They are located above damage fault zones, and have great practical interest for the assessment of radon hazard in populated areas.

The RAI values obtained in the study area provide a quantification of the geochemical activity of the PF system within the recognised TER zones. RAI values were grouped according to the classification procedure described in the RAI calculation section of the methods, and displayed as classed post map (Fig. 4).

Calculated RAI values (see Supplementary Table S2) range from 1.08 to 13.86 and are grouped in four classes according to the following classification^11^ scheme: low geochemical activity (RAI < 2), moderate geochemical activity (2 < RAI < 3), high geochemical activity (3 < RAI < 6) and very high geochemical activity (RAI > 6). The classed post map of the RAI values highlights that 25 fault-related radon concentration peaks out of 40 (about 62%) are localized in the fracture zone between the two main faults (PF and KV), most of the values falling in the moderate (9), high (5) and very high (5) geochemical activity groups; values below 2 only occur in the area of Chienes/Kiens municipality and to the south of the PF. The distribution of the RAI values in Fig. 4 describes a specific pattern which follows the fault strike direction and extends over the fracture zone which is 5 km wide to the east and 2 km wide to the west, where the two main faults PF and KV tend to converge.

High to very high RAI values mainly occur in the eastern sector (Falzes/Pfalzen municipality). In this area, the highest RAI values (mean value = 4.8) correspond to high radon concentration peaks aligned along the direction of minor faults in the middle of the TER area (dark grey zones in Fig. 4) between the PF and the DAV fault.

The western sector (Terento/Terenten municipality) displays medium to high RAI values (mean value = 2.9) and the associated radon peaks are strictly located within the TER area occurring between the PF and KV. In this sector, RAI values of the Rn peaks show a good spatial continuity as suggested by the variogram model, and the radon anomaly in this case is controlled by increased gas permeability of the damage zone along this fault segment of the PF.

The central sector (Chienes/Kiens municipality) displays low RAI values (mean value = 1.6). Although located within the TER zone (dark grey TER area in Fig. 4), this area is characterised by a patchy pattern of the radon anomalies with fault segments along the main structure of the PF with radon activity slightly above the background values (< 100 kBq·m^−3^). These fault segments, bordered by secondary NNE-SSW tending transfer faults with sinistral relay ramp geometry, are characterised by relatively unfractured rocks covered by weathered soil that lowers the gas permeability thus having a high impact on the intensity of radon anomalies near the fault zone. These conditions determine the patchy character of soil gas anomalies (including radon) at the surface (Fig. 1d), and confirm that in this sector of the valley the PF system, despite of the high fracture density (pale grey area in Fig. 4), gas permeability is highly variable along its entire extension^7,32^.

DAV mylonitic zone does not show any specific Rn peak pattern because it is characterised by a very low gas permeability due to its massive mylonitic microstructure. Furthermore, since this fault is located along the northern sector of the study area, boundary effects due to the low sample density makes the estimate less robust due to the lack of neighbouring points^37–39^. According to the distribution of the TER zones and their RAI values, the Pusteria fault system displays different distribution patterns of the anomalies and variable geochemical activity. In particular, in the western sector, where the different fault lines of the PF system tend to converge, the fault system behaves as a simple single fault (e.g., A and B in Fig. 1), whereas in the eastern sector the PF system acts as a complex fault system (e.g., C and D in Fig. 1). The distribution of the TER zones, explained in terms of RAI, confirms that they are characterised by high to very high radon values when associated with high fault/fracture density.

The relative RAI values obtained along different N-S profiles reflect the contrast between the radon anomalies and the background. According to the described data, RAI values can vary along fault strike within an order of magnitude depending on the type and fracture density of the fault zone. The individuation of contrasting anomalies and the calculation of the Radon Activity Index (i.e. RAI) is crucial to quantify TER in a fault zone and interpret the data in terms of geochemical activity. The obtained results are in line with the RAI calculated for some seismically active faults reported in the literature confirming that also non-active faults may increase the GRP (Table 2). We can establish that the collection of soil gas radon measurements for mapping the TER zones, as well as the calculation of RAI values in fault areas, are of practical interest for local authorities in the assessment of radon hazard, especially in urban areas.

**Table 2:** Values of RAI obtained by the ratio between the maximum value measured in case study the area and the background value over different areas in the world.

Author	Area	Fault type	Maximum value	Background value	RAI
This study	Pusteria valley —Periadriatic fault system	Transpressive fault	816 kBq m^−3^	50 kBq m^−3^	16
Ciotoli et al.^7^	Fucino plain—SBGMF	Normal fault	119 kBq m^−3^	25 kBq m^−3^	5
Zhou et al.^40^	Tibet—Lhasa	Normal fault	87.4 kBq m^−3^	7.6 kBq m^−3^	11
Li et al.^41^	Yanhuai basin, Hebei	undefined	57.8 kBq m^−3^	8.1 kBq m^−3^	7
Yao and Wang^42^	Jixian mountain, Tianjing	undefined	58.6 kBq m^−3^	3.2 kBq m^−3^	18
Zhou et al.^43^	Haiyuan, Ningxia	undefined	38.3 kBq m^−3^	5.8 kBq m^−3^	7
Seminsky et al.^11^	Baikal-Mongolian sesismic belt—Khustai fault	Normal fault	20.2 kBq m^−3^	3.9 kBq m^−3^	5
Wang et al.^16^	North China—Tangshan area	Normal fault	38.4 kBq m^−3^	4.7 kBq m^−3^	8
Chen et al.^13^	Capital of China—KQF fault	undefined	206.7 kBq m^−3^	11.6 kBq m^−3^	18
Xuan et al.^44^	Thua Thien Hue (Vietnam)—Dak Rong-Hue fault	undefined	144.5 kBq m^−3^	24.8 kBq m^−3^	6

## Conclusions

The main goal of this study was to evaluate the potential degassing processes along an aseismic fault system, the Pusteria fault system, in north-eastern Italy. To accomplish this objective, we have defined a further geogenic radon component (Tectonically Enhanced Radon, TER) that represents the contribution of tectonics to the Geogenic Radon Potential (GRP) of an area. Then, we have quantified the TER component by calculating the Radon Activity Index (RAI) that defines the geochemical activity of a fault in terms of Rn emission. The obtained results highlight the following conclusions:

- the fracture zone of the Pusteria fault system, including the main faults PF and KV, and other minor faults, plays a fundamental role for gas (^222^Rn and CO_2_) migration towards the surface thus providing TER as an additional contribution to the Rn diffusing from the source rocks (i.e., lithological background); both these components account for the GRP of an area;
- the summary map of the TER zones (Fig. 4) confirms that most of the areas characterised by high radon values are also associated with high fault/fracture density;
- radon activity index (RAI) calculated at Rn peak values along N-S profiles represents a good proxy to quantify the geochemical activity (e.g., gas-bearing properties) of a fault zone. We recognised that TER areas are characterised by medium to very high RAI values. However, although the central sector of the PF system shows high fault/fracture density, the low RAI values suggest the presence of less permeable segments along the fault system;
- the comparison of the calculated RAI with those calculated from seismic areas with active faults, reported in the literature, highlights that also aseismic faults can provide conduits for Rn migration towards the surface with the same order of magnitude of seismic faults;
- since the radon high activity generated from source rocks represents background areas of potential radon risk for inhabitants, the quantification and mapping of the TER areas are important to better evaluate the potential risk due to the increased radon availability to influx within buildings.
- the knowledge of these two components of the GRP is fundamental for the mapping of susceptibility of the territory at different spatial scales and in different geological scenarios, and can help policy makers to plan monitoring activities and take mitigation actions reducing the damage for the society. The future developments of TER concept will have important implications on the identification of radon priority areas (RPA) as required by the European Directive 59/2013^3^.

## Methods

### Structural model

**Figure 5:**
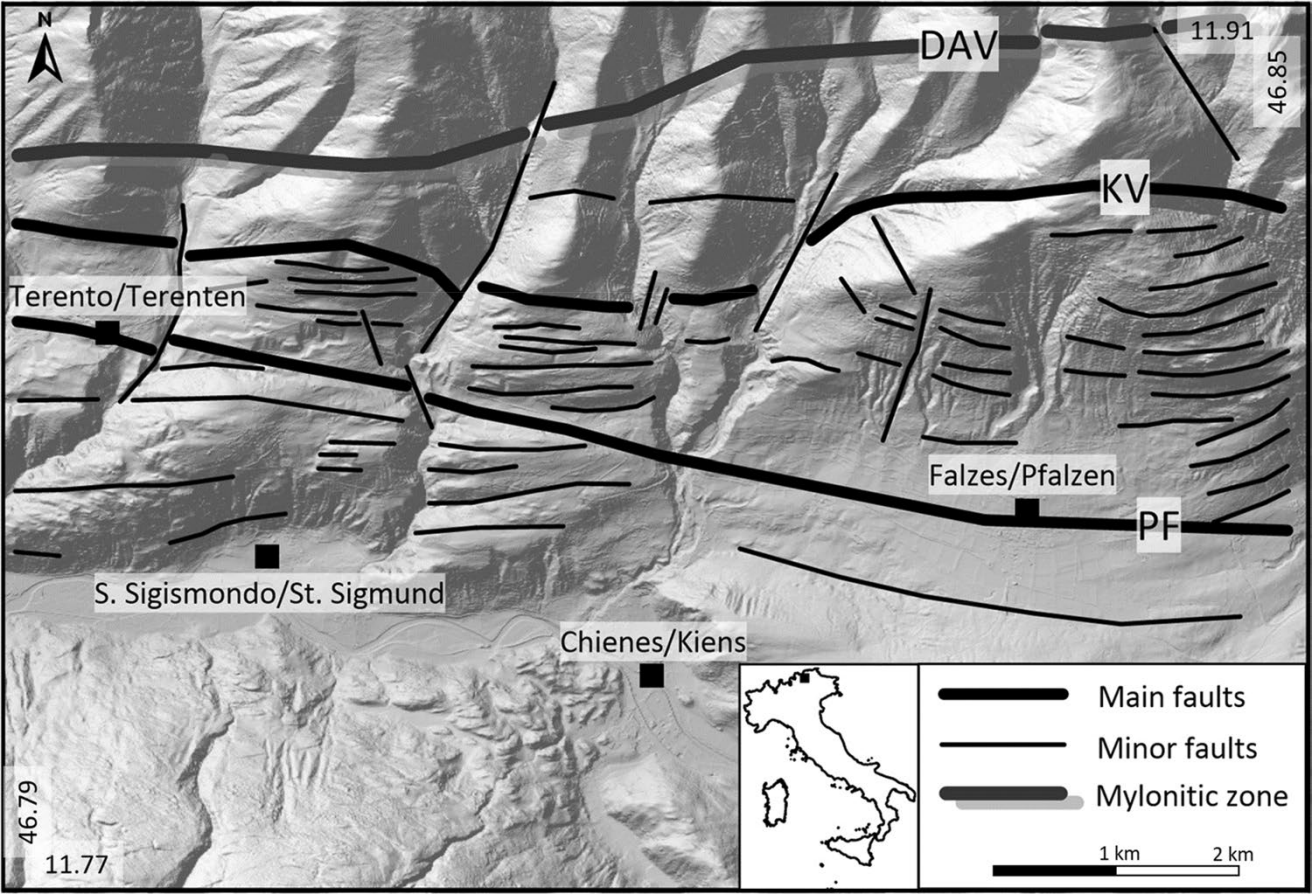
The structural model of the study area of the hillshade derived from the digital terrain model (DTM, 2.5 m/pixel). PF = Pusteria/Pustertal Fault; KV = Kalkstein-Vallarga/Weitental fault; DAV = Deffereggen-Anterselva/Antholz-Valles/Vals mylonitic zone.

The geological map^45^ and the location of the main faults^24^ are available from the Geological catalogue of Bolzano Province. The structure of the fault zone was reconstructed based on the geomorphological analysis of the Digital Terrain Model (DTM) at the resolution of 2.5 m. In particular, a detailed structural interpretation has been carried out on the hillshade derived from the DTM using ArcGIS Pro. We reclassified the recognised structures in three different classes: (*i*) main faults: including the PF and the KV faults; (*ii*) DAV: the mylonitic zone; (*iii*) minor faults (Fig. 5). The shapefile of the faults was then transformed in fault/fracture density map by using the Kernel Density algorithm^46^ of the Spatial Analyst tools in ArcGIS Pro.

### Radionuclide content

A total of 14 rock samples of the main outcropping lithologies in the study area were collected as follow: gneiss (7 samples) from the Austroalpine unit; phyllite (3 samples) and Brixen granite (4 samples) from the Southalpine unit. Prior to radioactivity measurement, the rock samples were ground and mechanically sieved (< 4 mm) to determine the activity concentration of natural radionuclides (^238^U, ^232^Th, ^40^ K) using high-resolution gamma-ray spectrometer in a suitable configuration. Rock samples were analysed using two p-type coaxial Hyper Pure Germanium crystal detector (HPGe), a PROFILE (Ortec-Ametek Inc.) with an extended energy range (20–2000 keV) and a GEM model (Ortec-Ametek Inc.) with an energy range 80–2000 keV. These detectors have relative efficiency of 20% and 38%, and resolution (FWHM) at 1322.5 keV of 1.9 keV and 1.8 keV, respectively. Both systems were calibrated for energy and efficiency using liquid standard solutions (Eckert and Ziegler Multi-nuclide standard solution 7501) in a jar geometry (diameter = 56 mm; thickness = 10 mm). Spectra of the rock samples were acquired for one day to optimize peak analysis and subsequently processed and analysed using the Gamma Vision-32 software package (version 6.07, Ortec-Ametek). ^226^Ra was determined at 186 keV correcting the peak area by the ^235^U interference according to the method proposed by^47^, under the hypothesis of secular equilibrium between ^226^Ra-^238^U and natural ^235^U/^238^U isotopic ratio. ^238^U and ^232^Th were then determined using the emission of their radioactive daughters ^226^Ra and ^228^Ac. Data validation and quality control was carried out analysing a series of certified reference materials (CRMs) in the same geometry as the unknown samples and calibration standard. The CRMs used, in the solid phase, were: IAEA-412 Pacific Ocean Sediment, UTS-3 (Canmet) and Dh1-a (Canmet). Minimum detectable activity (CR_MDA_) was determined using the Traditional ORTEC method (ORTEC, 2003) with a peak cut-off limit of 40%. Conversion from specific activity (Bq·kg^−1^) to bulk elemental weight fraction was obtained with the following conversion factors^48^:

$${1 }\% {\text{ K }} = { 3}0{9}.{\text{7 Bq}}\cdot{\text{kg}}^{{ - {1}}}$$

$${\text{1 ppm U }} = { 12}.{\text{35 Bq}}\cdot{\text{kg}}^{{ - {1}}}$$

$${\text{1 ppm Th }} = { 4}.0{\text{72 Bq}}\cdot{\text{kg}}^{{ - {1}}}$$

The radon background production (as well as the geochemical anomaly threshold) has been calculated using the ^222^Rn activity at equilibrium with parent radionuclides (^226^Ra) in collected rock samples, using the Akerblom formula^26^:

$$C_{Rn} = C_{Ra} \varepsilon \rho n^{ - 1}$$

where $C_{Rn}$ and $C_{Ra}$ are radon in soil gas (Bq·L^−1^) and radium in soil (Bq·kg^−1^), respectively, $\varepsilon$ is the emanation power coefficient (dimensionless), $\rho$ is soil density (kg·L^−1^), and $n$ is the effective porosity coefficient (dimensionless). The emanation power coefficient has been calculated as one minus the ratio between the mean activity concentration of radon decay products ^214^Pb and ^214^Bi with respect to the parent ^226^Ra^49,50^. We have assumed that $\rho$ = 2.7 g cm^−3^ and $n$ = 0.4.

### Soil gas sampling

Soil gas surveys were performed in July 2021 to ensure stable meteorological conditions. A total of 278 soil gas samples have been collected in an area of about 60 km^2^ according to a 500 m × 500 m grid and along 3 north–south profiles (P1, P2, P3) crossing the fault zone with a sampling step of about 100 m, in the municipalities of Terento/Terenten (western sector), Chienes/Kiens (central sector) and Falzes/Pfalzen (eastern sector).

We collected the soil gas samples using a 6.4 mm thick-walled stainless-steel probe pounded in the ground by a co-axial hammer at a depth of about 0.8–1 m to minimize as much as possible the influence of meteorological parameters that may affect the air exchange at the soil-atmosphere boundary. Soil gas measurements were conducted using a portable multi-gas analyser (Draeger X-am 7000, Drägerwerk AG&Co. KGaA) connected to the probe with a silicon tube for the simultaneous analyses of carbon dioxide (CO_2_, range 0–100%), methane (CH_4_, range 0–100% LEL), hydrogen (H_2_, range 0–600 ppm), hydrogen sulfide (H_2_S, range 0–1000 ppm) and oxygen (O_2_, range 0–21%). Radon (^222^Rn) and thoron (^220^Rn) measurements were conducted using RAD7 (Durridge Company, Inc) alpha detector (± 5% absolute accuracy, and a sensivity of 0.25 cpm/(pCi/L) 0.0067 cpm/(Bq·m^−3^). Each measurement of radon and thoron activity is performed with 5-min integration time, and is repeated until the difference between the last two measurements is at least below 5–10%. The final result was determined by taking the average of the last two integrations^32^.

### Statistical and geospatial analyses

Collected data have been processed using: Exploratory Data Analysis (EDA) and Geostatistical Analysis (GA).

EDA was performed to evaluate the basic characteristics of the data and their statistical distribution by using numerical (i.e., calculation of summary statistics and statistical distribution of each variable) and graphical methods (i.e., histograms, box plots and normal probability plots). In particular, we used the Normal Probability Plots (NPP) to determine the occurrence of different overlapping geochemical populations and define threshold values by approximating linear segments on the point distribution in the graph^49^. The identification of sharp deviations or gaps in the NPP may indicate the presence of subpopulations (e.g., background and/or anomalies) separated by a threshold value^51–53^.

GA was performed to understand and reconstruct the natural phenomena that govern the spatial behaviour of the studied variables. In particular, we used GA to visualize the samples distribution and the distribution of the measured values compared to their nearest neighbours, to study the spatial autocorrelation of the variables and elaborate a spatial model in order to estimate the variable values at unsampled locations and construct final prediction maps.

We accomplished this process according to the following steps:

1. Construction of experimental variograms of the studied variables. In particular experimental variograms and variogram surfaces were used to check the spatial continuity of the data distribution values and the presence of anisotropic phenomena (i.e., fault-related) acting along preferential directions;
2. Determination of the anisotropy (where present) which is important for defining parameters for the kriging estimation (i.e., directions and anisotropy ratio);
3. Construction of a spatial model, i.e., calculation of the main variogram parameters (nugget, range, sill) to be used in the kriging algorithm;
4. Preparation and validation of prediction maps (i.e., contour maps) by using ordinary kriging (OK) algorithm^25^.

Collected data were processed using the following software: ArcGIS Pro 2.7.0 (copyright 2020@Esri Inc.) and Surfer 23.1.162 (copyright 1993–2021, Golden Software, LLC) for the mapping process; Grapher 19.1.288 (copyright 1992–2021, Golden Software, LLC) and Statistica 12 (copyright Statsofts. Inc.) software for the numerical and graphical statistics.

### RAI calculation

To quantify the Tectonically Enhanced Radon (TER) we have applied the concept of Radon Activity Index (RAI). Over the years, many systems for classification radon concentration in soil gas have been elaborated to mathematically explain the geochemical activity of a fault zone. Seminsky et al.^11^ proposed the relative index of radon activity K_Q_ calculated as the ratio of the maximum ^222^Rn concentration (Q_max_) to the minimum ^222^Rn concentration outside the fault zone (Q_min_) for the classification of fault activity. Then, the values have been divided into five different levels of activity.

Inspired by this study, we have modified the concept of the index of radon activity, considering a Radon Activity Index (RAI) calculated as the ratio between the maximum ^222^Rn value along the measured or estimated radon profiles to the background value (50 kBq·m^−3^) estimated over the area with the NPP method (see section geostatistical and spatial analysis in methods).

We have calculated the RAI considering only the radon peaks (radon values above the background threshold) relative to 21 (P4 to P24) estimated profiles, obtained by intersecting the radon grid map with 6 km long N-S sections (using Profile, Map Tools in Surfer 23.1.162) with a sampling step of 10 m and spaced 500 m from each other. Applying this method, each profile is composed of 600 estimated points of measurement ensuring good statistic representativeness. Based on the results obtained by RAI calculation, the values have been divided into four classes of geochemical activity: (i) RAI < 2: low activity, (ii) 2 < RAI < 3: medium activity; (iii) 3 < RAI < 6 high activity; (iv) RAI > 6 very high activity (see Supplementary Table S2).

The location of the RAI values is reported as classed post in a bivariate colour map (summary sketch map in Fig. 4) obtained using the bivariate colours option in ArcGIS Pro. The different colours in the bivariate map represent different values of the first (RAI) and the second (fault density) variable simultaneously.

## Supplementary Information

Supplementary Information 1.

## Data Availability

All data generated or analysed during this study are included in this article (and its Supplementary Information files).
