# Supplementary material for: Evaluation of tectonically enhanced radon in fault zones by quantification of the radon activity index
Source: Sci Rep. 2022 Dec 14;12:21586. doi: 10.1038/s41598-022-26124-y (PMC9751298; doi:10.1038/s41598-022-26124-y)
Supplement: Supplementary file 1 — Supplementary Information 1. [file 41598_2022_26124_MOESM1_ESM.docx]

**Evaluation of Tectonically Enhanced Radon in fault zones by quantification of the Radon Activity Index**

**Authors and affiliations:**

Eleonora Benà^1*^, Giancarlo Ciotoli^2,3*^, Livio Ruggiero^3^, Chiara Coletti^1^, Peter Bossew^4^, Matteo Massironi^1^, Claudio Mazzoli^1^, Volkmar Mair^5^, Corrado Morelli^5^, Antonio Galgaro^1^, Pietro Morozzi^6^, Laura Tositti^6^, Raffaele Sassi^1^

^1^ Dipartimento di Geoscienze, Università di Padova, Via Gradenigo 6, 35131 Padova, Italy. eleonora.bena@phd.unipd.it

^2^ Istituto di Geologia Ambientale e Geoingegneria (IGAG), Consiglio Nazionale delle Ricerche (CNR), 00015 Monterotondo, Rome, Italy. giancarlo.ciotoli@igag.cnr.it

^3^ Istituto Nazionale di Geofisica e Vulcanologia (INGV), Via di Vigna Murata 605, 00143 Rome, Italy

^4^ Retired from Federal Office for Radiation Protection (BfS), Section Radon and NORM, Köpenicker Allee 120-130, 10318 Berlin, Germany

^5^ Provincia Autonoma di Bolzano, Ufficio Geologia e Prove Materiali, Cardano-Kardaun, Italy

^6^ Dipartimento di Chimica “G. Ciamician”, Università di Bologna, Via Selmi 2, 40126 Bologna, Italy

*corresponding authors: eleonora.bena@phd.unipd.it, giancarlo.ciotoli@igag.cnr.it

**Figure S1:**


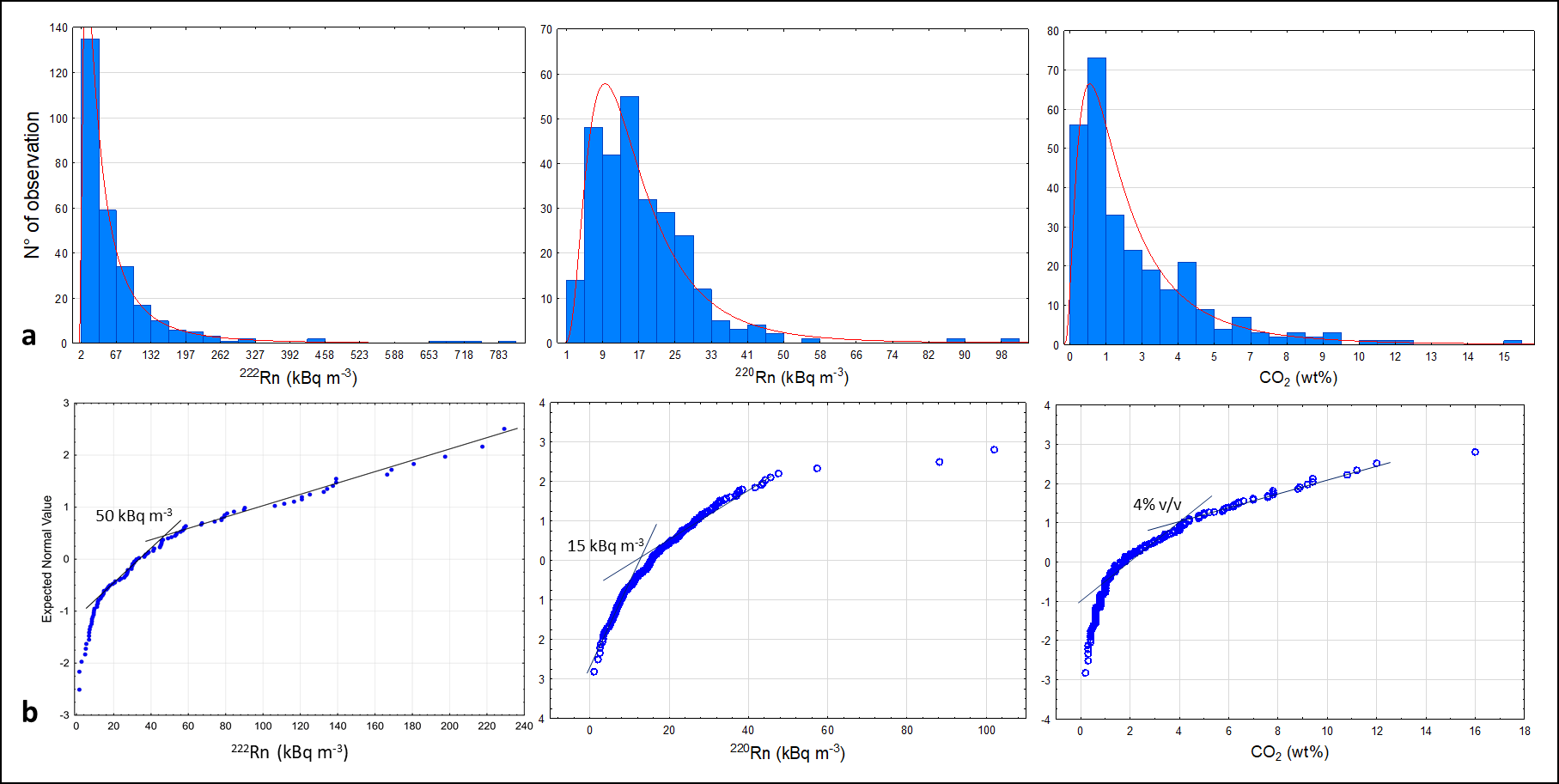


Figure S1. (a) Histograms and (b) normal probability plots and of soil gas data. Concerning the NPP of radon, the x axis ranges between 1.54 kBq·m^-3^ (min) and 240 kBq·m^-3^, in order to better highlight the anomaly threshold.

**Table S1:**

| Lithotype |  | ^238^U | σ % | ^226^Ra | σ % | ^232^Th | σ % | ^40^K | σ % | U | Ra | Th | K | ^222^Rn* |
| --- | --- | --- | --- | --- | --- | --- | --- | --- | --- | --- | --- | --- | --- | --- |
|  |  | (Bq kg^-1^) |  | (Bq kg^-1^) |  | (Bq kg^-1^) |  | (Bq kg^-1^) |  | (ppm) | (ppm) | (ppm) | (wt%) | (Bq m^-3^) |
| Gneiss | PU04 | 19 | 18 | 82 | 13 | 38 | 11 | 1469 | 6 | 1.5 | 7.4 | 9.4 | 4.7 | 83.50 |
|  | AN01 | 22 | 19 | 60 | 11 | 42 | 16 | 1436 | 6 | 1.8 | 5.4 | 10.3 | 4.6 |  |
|  | CS01 | 21 | 22 | 25 | 17 | 26 | 9 | 627 | 6 | 1.7 | 2.3 | 6.4 | 2.0 |  |
|  | PU02 | 49 | 16 | 59 | 12 | 40 | 19 | 1025 | 6 | 4.0 | 5.4 | 9.7 | 3.3 |  |
|  | NE02 | 15 | 28 | 19 | 27 | 26 | 14 | 695 | 6 | 1.2 | 1.7 | 6.5 | 2.2 |  |
|  | NE03 | 33 | 11 | 41 | 9 | 50 | 7 | 689 | 6 | 2.6 | 3.7 | 12.3 | 2.2 |  |
|  | FA01 | 26 | 12 | 26 | 18 | 57 | 7 | 1325 | 5 | 2.1 | 2.3 | 14.1 | 4.2 |  |
| Granite | MU05 | 47 | 20 | 75 | 12 | 111 | 9 | 1575 | 6 | 3.8 | 6.8 | 17.2 | 5.0 | 68.30 |
|  | FA03 | 38 | 20 | 41 | 11 | 69 | 9 | 428 | 7 | 3.0 | 3.7 | 16.9 | 1.4 |  |
|  | FA04 | 25 | 26 | 28 | 23 | 47 | 11 | 942 | 6 | 2.1 | 2.5 | 11.5 | 3.0 |  |
|  | FA05 | 35 | 17 | 51 | 9 | 52 | 7 | 1121 | 6 | 2.9 | 4.6 | 12.9 | 3.6 |  |
| Phyllite | PU05 | 26 | 15 | 24 | 14 | 32 | 8 | 436 | 7 | 2.1 | 2.1 | 7.8 | 1.4 | 27.71 |
|  | SS01 | 25 | 10 | 35 | 12 | 52 | 8 | 1100 | 5 | 2.0 | 3.1 | 12.7 | 3.5 |  |
|  | SS02 | 36 | 15 | 49 | 20 | 87 | 7 | 1352 | 5 | 2.9 | 4.4 | 21.3 | 4.3 |  |

Table S1. Radionuclide content (Bq·kg^-1^) in the main outcropping lithologies in the study area: gneiss (e.g., orthogneiss, paragneiss), granite and phyllite. *^222^Rn = mean ^222^Rn activity (kBq·m^-3^) at equilibrium with parent radionuclides (^226^Ra) obtained by the Akerblom formula^26^.

**Figure S2:**

**
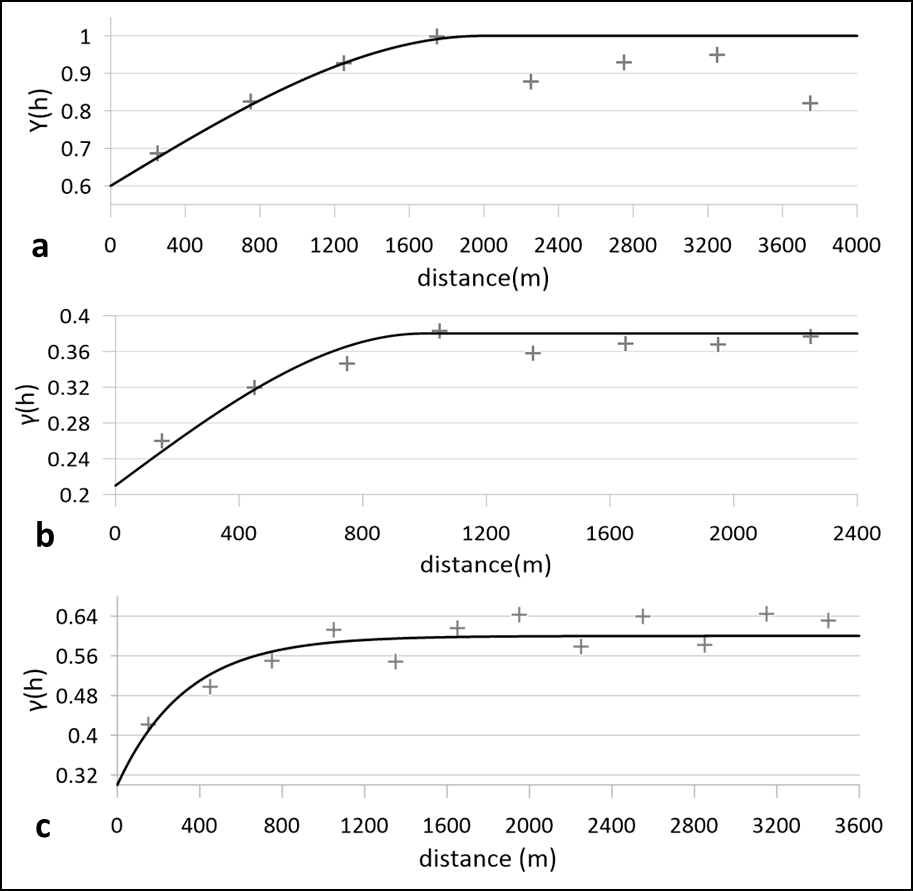
**

Figure S2. Experimental variograms and models calculated for (a) ^222^Rn, (b) ^220^Rn and (c) CO_2_. Crosses indicate mean γ values for each lag and continuous lines indicate the variogram models. Following the equations of the selected models: γ(h) = 0.6*Nugget+0.4*Spherical (2000, 1000, 90) for ^222^Rn (a); γ(h) = 0.21*Nugget+0.17*Spherical (1000, 500, 50) for ^220^Rn (b); γ(h) = 0.3*Nugget+0.3*Exponential (1000, 500, 95) for CO_2_ (c).

**Figure S3:**


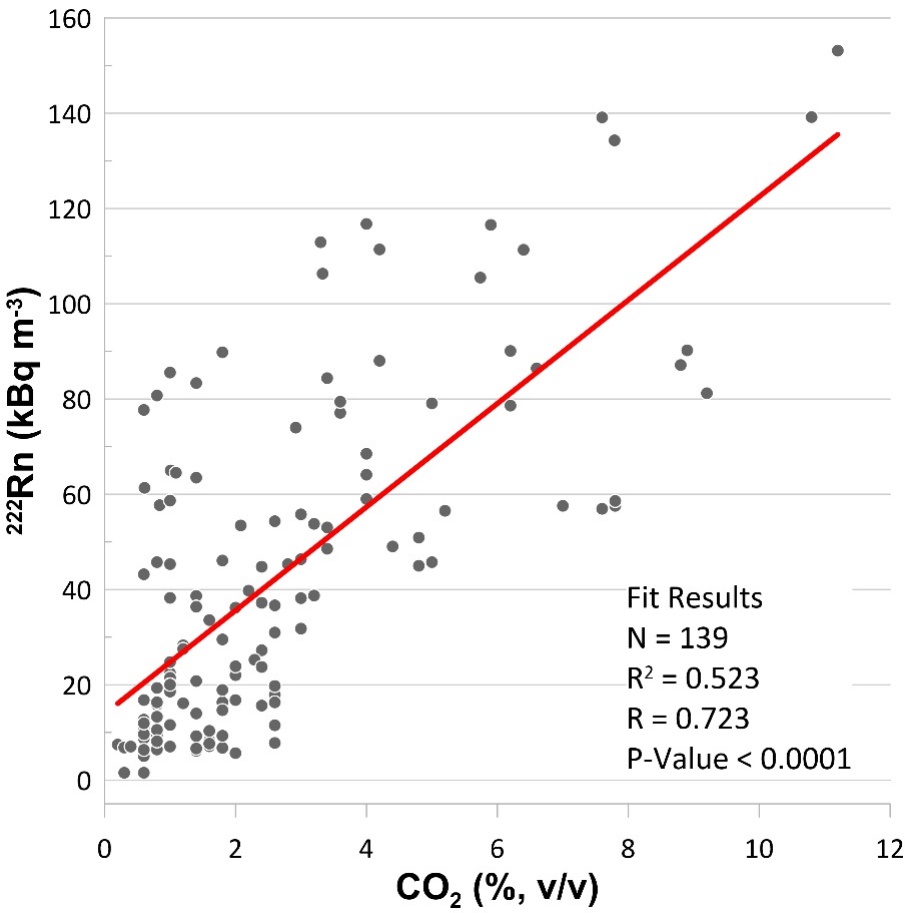


Figure S3. Scatterplot ^222^Rn (kBq·m^-3^) versus CO_2_ (%, v/v) shows that there is a significant linear relationship between the two gases (p value < 0.0001) with coefficients R = 0.723 and R^2^ = 0.523.

**Table S2:**

| Profile | N. peak | X | Y | Rn max | RAI |
| --- | --- | --- | --- | --- | --- |
| P4 | 1 | 712003 | 5189508 | 57 | 1.14 |
| P5 | 1 | 712513 | 5190538 | 109 | 2.18 |
|  | 2 | 712513 | 5188437 | 68 | 1.36 |
| P6 | 1 | 713003 | 5190406 | 243 | 4.86 |
| P7 | 1 | 713502 | 5190416 | 122 | 2.44 |
| P8 | 1 | 714012 | 5189998 | 193 | 3.86 |
| P9 | 1 | 714512 | 5189987 | 134 | 2.68 |
| P10 | 1 | 715002 | 5189998 | 90 | 1.80 |
| P11 | 1 | 715502 | 5189559 | 120 | 2.40 |
|  | 2 | 715502 | 5188580 | 56 | 1.12 |
| P12 | 1 | 716001 | 5189804 | 68 | 1.36 |
|  | 2 | 716001 | 5188825 | 79 | 1.58 |
| P13 | 1 | 716491 | 5189814 | 74 | 1.48 |
| P14 | 1 | 717001 | 5190344 | 54 | 1.08 |
|  | 2 | 716991 | 5188886 | 74 | 1.48 |
|  | 3 | 717511 | 5190518 | 91 | 1.82 |
|  | 4 | 717501 | 5188539 | 65 | 1.30 |
| P16 | 1 | 718001 | 5189712 | 115 | 2.30 |
| P17 | 1 | 718500 | 5190508 | 69 | 1.38 |
|  | 2 | 718500 | 5188702 | 110 | 2.20 |
| P18 | 1 | 719000 | 5191619 | 69 | 1.38 |
|  | 2 | 719000 | 5189579 | 132 | 2.64 |
| P19 | 1 | 719510 | 5191130 | 693 | 13.86 |
|  | 2 | 719500 | 5189987 | 125 | 2.50 |
|  | 3 | 719500 | 5188570 | 288 | 5.76 |
| P20 | 1 | 720000 | 5191925 | 188 | 3.76 |
|  | 2 | 720010 | 5190069 | 159 | 3.18 |
|  | 3 | 719990 | 5188886 | 135 | 2.70 |
|  | 4 | 720010 | 5187907 | 124 | 2.48 |
| P21 | 1 | 720500 | 5191915 | 119 | 2.38 |
|  | 2 | 720500 | 5189916 | 392 | 7.84 |
|  | 3 | 720510 | 5188172 | 174 | 3.48 |
| P22 | 1 | 721010 | 5191762 | 151 | 3.02 |
|  | 2 | 720999 | 5189926 | 611 | 12.22 |
| P23 | 1 | 721499 | 5191997 | 112 | 2.24 |
|  | 2 | 721499 | 5191007 | 499 | 9.98 |
|  | 3 | 721489 | 5190008 | 308 | 6.16 |
|  | 4 | 721510 | 5189008 | 159 | 3.18 |
| P24 | 1 | 721999 | 5190997 | 213 | 4.26 |
|  | 2 | 721999 | 5188865 | 115 | 2.30 |

Table S2. Radon Activity Index (RAI) values calculated in correspondence of radon peaks along the estimated (from P4 to P24) profiles in the study area. N. peak = number of considered peaks along the i^th^ profile; X, Y = location of the radon peaks; Rn max = maximum radon value along the i^th^ profile; RAI = Radon Activity Index calculated as the ratio between the maximum radon value and the background value (50 kBq·m^-3^).
